# A Novel High Glucose-Tolerant β-Glucosidase: Targeted Computational Approach for Metagenomic Screening

**DOI:** 10.3389/fbioe.2020.00813

**Published:** 2020-07-30

**Authors:** Shohreh Ariaeenejad, Safura Nooshi-Nedamani, Mahdie Rahban, Kaveh Kavousi, Atefeh Ghasemi Pirbalooti, SeyedSoheil Mirghaderi, Mahsa Mohammadi, Mehdi Mirzaei, Ghasem Hosseini Salekdeh

**Affiliations:** ^1^Department of Systems and Synthetic Biology, Agricultural Biotechnology Research Institute of Iran (ABRII), Agricultural Research Education and Extension Organization (AREEO), Karaj, Iran; ^2^Laboratory of Complex Biological Systems and Bioinformatics (CBB), Department of Bioinformatics, Institute of Biochemistry and Biophysics (IBB), University of Tehran, Tehran, Iran; ^3^Department of Molecular Sciences, Macquarie University, Sydney, NSW, Australia

**Keywords:** novel β-glucosidase, *in silico* screening, lignocellulosic biomass, high glucose tolerance, metagenome

## Abstract

The rate-limiting component of cellulase for efficient degradation of lignocellulosic biomass through the enzymatic route depends on glucosidase’s sensitivity to the end product (glucose). Therefore, there is still a keen interest in finding glucose-tolerant β-glucosidase (BGL) that is active at high glucose concentrations. The main objective of this study was to identify, isolate, and characterize novel highly glucose-tolerant and halotolerant β-glucosidase gene (PersiBGL1) from the mixed genome DNA of sheep rumen metagenome as a suitable environment for efficient cellulase by computationally guided experiments instead of costly functional screening. At first, an *in silico* screening approach was utilized to find primary candidate enzymes with superior properties. The structure-dependent mechanism of glucose tolerance was investigated for candidate enzymes. Among the computationally selected candidates, PersiBGL1 was cloned, isolated, and structurally characterized, which achieved very high activity in relatively high temperatures and alkaline pH and was successfully used for the hydrolysis of cellobiose. This enzyme exhibits a very high glucose tolerance, with the highest inhibition constant *K*_*i*_ (8.8 M) among BGLs reported so far and retained 75% of its initial activity in the presence of 10 M glucose. Furthermore, a group of multivalent metal, including Mg^2+^, Mn^2+^, and Ca^2+^, as a cofactor, could improve the catalytic efficiency of PersiBGL1. Our results demonstrated the power of computational selected candidates to discover novel glucose tolerance BGL, effective for the bioconversion of lignocellulosic biomass.

## Introduction

The term cellulosic biomass, also known as lignocellulose, refers to any constituent of plants or plant-derived biodegradable matter, namely, wood, grasses, and other feedstock across the world. Lignocellulose is mainly composed of three structural polymers (cellulose, hemicellulose, and lignin). It is the most prevalent type of raw material that exists on the earth (Guo et al., 2018). The enormous quantity of cellulosic biomass and its renewable quality have led to a strong drive for replacing fossil fuel with more eco-friendly energy sources.

The use of fossil fuel and the problems associated with it, such as contributing to environmental pollution and the fact that nature cannot replenish fossil energy sources, have forced us to forage for an alternative and renewable source of energy (Singhania et al., 2017). In this respect, bioethanol production is receiving abundant consideration as a promising candidate for biofuel, and hence substances that participate in biofuel production are of great economic importance (Zhang et al., 2012).

Enzymatic saccharification of cellulose in biofuel production usually depends on at least three enzymes, including endoglucanases (EGs), cellobiohydrolases (CBHs), and β-glucosidases (BGLs). Together, the coordinated action of these three enzymes induces a synergistic effect on cellulose degradation. EGs hydrolyze the long-chain cellulose into cellodextrin and oligosaccharides. CBHs release cellobiose units, and in the end, BGLs play a pivotal role in converting cellobiose into glucose (Cairns and Esen, 2010; Cao et al., 2015).

β-glucosidases are a set of enzymes that are typically classified in GH1 and GH3 families, with less presence in GH families (Chan et al., 2016; Cao et al., 2018) 5, 9,16, 30, and 116 (Thongpoo et al., 2013). BGL is involved in the process of glycosidic bond cleavage that ends in glucose release. Therefore, BGLs are essential for the efficiency and completion of cellulose hydrolysis (Colabardini et al., 2016) and likewise determining the rate of forwarding reactions (Santa-Rosa et al., 2018). BGLs have been exploited in a broad range of applications such as food, animal feed, beverage, biofuel industries, and many other commercial fields (Pogorzelski and Wilkowska, 2007; Nigam et al., 2018). In the past decade, by the advent of biofuel and biomass program, BGL has drawn thorough attention for its function in the final step of lignocellulose bioconversion (Cai et al., 2019).

The catalytic efficiency of BGL is undergoing considerable interest, reasoning that its ultimate product, glucose, inhibits and limits the activity of BGL, which leads to cellobiose and oligosaccharide aggregation. Subsequently, the presence of an accumulated concentration of cellobiose and oligosaccharide operates as a barrier to repress endoglucanase and exoglucanase, which eventually hinders the whole progress of cellulose hydrolysis (Singhania et al., 2013; Teugjas and Väljamäe, 2013).

In industrial bioconversion, reaction conditions, e.g., temperature and pH stability, substrate concentration, and inhibitors are remarkable factors that can affect the hydrolyzed quality of BGLs (Limauro et al., 2018; Cai et al., 2019). Regarding the industrial utilization of enzymes that often occurs at a specific range of temperature (50°C) for an extended period and the fact that most of the BGLs with glucose resistance such as BGLs acquired from *Debaryomyces hansenii* and *Thermoanaerobacterium aotearoense* metagenome are heat-sensitive, the thermal stability of an enzyme is of great importance (Maitan-Alfenas et al., 2014; Yang et al., 2015). Thermostable enzymes offer numerous potential advantages in lignocelluloses bioconversion (Littlechild, 2015), such as increasing the reaction rate, the high solubility of the reactants, a lower risk of contamination, lowering the solution viscosity, and decreasing the cost of energy for cooling after thermal pretreatment (Seo et al., 2015; Zeiske et al., 2016). In 2017, it was proposed that thermostability, high catalytic efficiency, and a strong tolerance for glucose of BGLs are significant features for their industrial utilization (Pang et al., 2017).

Besides the properties as mentioned above, e.g., thermostability and glucose tolerance, sometimes enzymes based on their biotechnological and commercial purposes are required to have some peculiar features, for example, salt or metal ion tolerance. To the best of our knowledge, there is a dearth of information about halophilic BGLs reported in the literature hitherto, but a BGL from a marine streptomycete and another BGL from a marine metagenomic library are some examples of enzymes with salt tolerance characteristic (Fang et al., 2010; Mai et al., 2013).

In order to assess BGL sensitivity to glucose, two kinetic values could be assessed: The half-maximal inhibitory concentration of glucose (IC_50_) or inhibition constant (*K*_*i*_). By postulating the recent research, GH1 and GH3 BGLs have revealed several fluctuations in their glucose tolerance rate (Chauve et al., 2010). Nevertheless, the BGL that belongs to the GH1 family demonstrated much higher glucose tolerance in comparison to GH3 β-glucosidases (Thongpoo et al., 2014). Although scientists have not reached a consensus about the factors responsible for high glucose tolerance of GH1 β-glucosidases, some, including Giuseppe et al., associate the high glucose-tolerant GH1 BGLs with the deeper catalytic pocket of them (De Giuseppe et al., 2014). One of the most comprehensive investigations to perceive the structural mechanism behind high glucose tolerance in BGLs has been done by Matsuzawa et al. (2016). They showed that the asparagine residue located at position 223 of a high glucose-tolerant GH1 β-glucosidase, Td2F2, has a crucial role in the glucose tolerance feature. They examined the importance of the structure at the Asn223 residue by saturation mutagenesis via replacing it with all other amino acid residues and measured their activities in the presence or absence of 500 mM glucose. This mutation drastically reduced the enzyme glucose tolerance and its substrate specificity (Matsuzawa et al., 2016).

The isolation and biochemical characterization of BGLs from a widespread class of organisms have been studied for discovering novel and industrially more productive enzyme (Tramontina et al., 2017). Up to date, there are only four BGLs with both high thermostability and glucose tolerance that are directly acquired from a hyperthermophilic microorganism (Dennett and Blamey, 2016). However, their optimum working temperature was much higher than commercially used BGLs (Akram et al., 2016). To improve the industrial operation of BGLs and combine various expedient traits, genetically manipulated enzymes are used. For instance, the Cel1A mutant (167/172) with mended glucose tolerance and resistance against extreme condition as a result of two amino acid modification developed a better enzymatic source in bioconversion of lignocellulosic materials (Guo et al., 2016). Another example of genetically enhanced BGL is Bgl1317, which appears to be the first enzyme of its kind that performs acceptable thermostability and glucose tolerance (Liu et al., 2019).

Likewise, metagenomics approaches provide a new strategy that enabled us to clone experimentally unavailable genome into culturable bacteria to detect a unique biocatalyst. A new glucose-tolerant BGL (bgl1A) was isolated from the marine metagenome that showed 50% of its activity in 1 M glucose. This result demonstrates the feasible application of bgl1A in the degradation of biomass (Fang et al., 2010).

A high glucose-tolerant GH1 β-glucosidase, Td2F2, and Ks5A7 were isolated by functional screening from the microbial metagenomic library. The recombinant β-glucosidase, Td2F2 and Ks5A7, exhibited enzymatic activity with the product inhibition by glucose and is a potent candidate for industrial applications (Uchiyama et al., 2013, 2015).

In the current study, the sheep rumen metagenomic data were computationally screened to find out proper thermostable, halotolerant, and glucose-tolerant BGL candidate enzymes, and the PersiBGL1 was cloned, expressed, purified, and biochemically characterized. In this context, the biochemical feature of the enzyme, including optimum temperature and pH, thermostability, and its activity in the presence of glucose inhibitors and metal ions, were assayed and brought in the following section. The distinctive enzymatic properties of this enzyme revealed that it is appropriate for effectively degrading lignocellulosic biomass and can be potentially utilized in bioethanol production as well as other applications in food and feed industries.

## Materials and Methods

### Computational Mining of Thermostable, Halotolerant, and Glucose-Tolerant β-Glucosidase Sequences

Metagenomic data and the analysis protocol of sheep.s rumen microbiota (Supplementary File S1) were submitted to NCBI with BioProject ID: PRJNA635543. Raw sheep rumen metagenomic data were mined for putative glucose-tolerant and preferably halotolerant and/or thermostable BGL enzymes. After quality control by FASTQC and performing assembly process using meta-velvet assembler, the MetaGeneMark software (Zhu et al., 2010) was exploited to predict the β-glucosidase genes from constructed contigs. The enzymes extracted from the metagenome were aligned against the list of 13 BGL enzymes extracted from the literature using standalone NCBI BLAST (Supplementary File S2). The one common property among all selected enzymes is that they all have at least one of these experimentally proven features: glucose tolerance, halotolerant, thermophilicity, or alkaliphilicity.

Based on pairwise blast, the most similar predicted metagenomic BGLs to the abovementioned known enzymes, were determined with an E-value less than 1E-50.

To determine the evolutionary position of the chosen predicted enzyme, named PersiBGL1, the phylogenetic tree consisting of PersiBGL1 and 13 abovementioned known enzymes were inferred using the Neighbor-Joining method (Saitou and Nei, 1987).

For inferring the phylogenetic tree, the tree is drawn to scale, with branch lengths in the same units as those of the evolutionary distances used. The evolutionary distances were computed using the number of differences method (Nei and Kumar, 2000) and are in the units of the number of amino acid differences per sequence. There were a total of 321 positions in the final dataset. Evolutionary analyses were conducted in MEGA X (Kumar et al., 2018). One of the metagenomic enzymes, later called PersiBGL1, showed the most phylogenetic similarity with the known enzymes, after the following extra stages, and were selected for the lab experiments.

Additional computational steps were performed to narrow down the set of candidate metagenomics enzymes. The predicted enzymes from metagenome were compared with conserved domains using the PSSM models in the NCBI Conserved Domains Database (CDD) (Marchler-Bauer et al., 2016).

Among sequences with close similarity with known thermostable BGLs and predicted BGL CDD domain, the PersiBGL1 was chosen for further analysis. Using the Phyre2 server, the tertiary structure of this enzyme is predicted (Kelley et al., 2015). The predicted structure for PersiBGL1 was aligned against the Td2F2 as a high glucose-tolerant BGL with 3WH5 pdb code using the TM-align tools (Zhang and Skolnick, 2005).

### Recombinant Expression and Purification PersiBGL1

In order to acquire BGL gene, metagenome DNA template from sheep rumen was screened. The BGL gene encoding sequence was amplified by polymerase chain reaction (PCR) using PersiBGL1 F (5′-TAATAGCATATG ATGGCAGTCAAGTATCAGTTC-3′) and PersiBGL1 R (5′-TAATAG AAGCTT TCAAAATCCATGATTCGCAATG-3′) primers, which included *Nde*I and *Hin*dIII restriction sites, respectively. The PCR was carried out as follows: one cycle at 95°C for 5 min, 35 cycles of 94°C for 40 s, 55°C for 45 s, 72°C for 2 min, and then a cycle of 72°C for 10 min. PCR product was purified, digested with *Nde*I and *Hin*dIII, and ligated into pET-28a, resulting in recombinant plasmid pet28-PersiBGL1 (Ariaeenejad et al., 2019a, b).

The resulting PCR products were detected on agarose gel 1.5% (w/v) and purified using the gel extraction kit (BIORON, Germany). Purified DNA fragments were cloned and digested into the pET28an. The resulting plasmids were then transformed into the *E. coli* BLrad21 (DE3) and correct insertion was confirmed by sequencing. In the Luria-Bertani (LB) medium, the recombinant strain pET28-PersiBGL1 was cultivated at the temperature of 37°C. Adding isopropyl-β-D-thiogalactopyranoside (IPTG) to a final concentration of 0.4 mM for 20 h at 20°C, expression of enzymes was induced. By utilizing Ni-NTA Fast Start Kit (Qiagen, Hilden, Germany), N-terminal histidine-tagged recombinant protein was purified and evaluated by sodium dodecyl sulfate-polyacrylamide gel electrophoresis (SDS-PAGE).

The candidate enzyme produced from successful cloning, expression, and purification was named PersiBGL1. This BGL was subjected to further biophysical experiments. The nucleotide sequence of the GH1 gene PersiBGL1 was submitted to GenBank Database with accession number MN016943. The nucleotide and amino acid sequences of PersiBGL1 are available in Supplementary File S3.

Protein concentrations were determined through the Bradford method using bovine serum albumin as the standard.

### Spectroscopy Studies

Synergy HTX Multi-Mode Microplate Reader was used for UV–vis spectrophotometry. Bovine serum albumin (BSA) was used as a protein standard to matured protein concentration using the Bradford method. The β-pNPG substrate was used for enzyme activity measurement at 405 nm.

### Enzymatic Activity Analysis of β-Glucosidase

PersiBGL1 activity was examined through the micro titer plate method by employing 4-nitrophenyl-β-D-glucopyranoside (β-pNPG) as a substrate (Parry et al., 2001). The reaction systems containing 30 μl of β-pNPG (10 mM, pH 7) substrate and 30 μl of the appropriately diluted enzyme (0.5 mg/ml) were combined at 40°C for 10 min, and the reaction was terminated by adding 120 μl of Na_2_CO_3_ (1 M). By monitoring the absorbance at 405 nm, we recorded the absorbance of the developed color.

The standard curve of p-nitrophenol (pNP) has been used to measure the concentration of produced pNP. One unit of BGL was defined as the amount of enzyme required for releasing 1 μmol of pNP per minute under the specified conditions of these assays. The kinetic parameters Michaelis constant (*k*_*m*_ and *V*_*max*_) of PersiBGL1 was determined by the Lineweaver–Burk plot obtained from enzymatic hydrolysis of pNPG differing in concentration (from 0.1 to 10 mM, pH 7) (Chan et al., 2018).

### Determination of Optimum Temperature and pH and Storage Stability

The enzyme’s optimum temperature was identified by analyzing enzyme catalytic activity using β-pNPG (10 mM, pH 7) in temperatures varying between 20 and 90°C, during 10 min incubation of reaction mixture at each temperature and also the enzyme’s optimal pH was similarly determined by investigating enzyme activity in the range of 4 to 11 at optimum temperature. The optimal reaction pH was assessed using several buffers with varying pH values at 40°C including citrate sodium buffer (50 mM, pH 4.0, 5.0), phosphate buffer (50 mM, pH 6.0–8.0), Tris–HCl buffer (50 mM, 9), and carbonate-bicarbonate buffer (pH 10, 11).

The storage stability of enzyme was determined by pre-incubation of the purified enzyme. The methods for determining storage stability was used by incubation of the purified enzymes for 11 days in elution buffer Ni-NTA Fast Start Kit (Tris–HCl 5 mM, NaCl 300 mM, and imidazole 250 mM, at pH 8.0) at 40°C, and the enzymes’ activities were examined in each 24-hour interval. For reporting PersiBGL1 activity, relative activity was considered as the percentage of highest activity.

### Glucose Inhibition Studies

To investigate the effect of the end product, glucose, on the catalytic activity, this reaction was performed in the presence of glucose concentration from 0 to 10 M. The concentration of the initial substrate pNPG was 10 mM. For the control, the same reaction system was used, but no glucose was added. The inhibitory influence of glucose on the initial enzymatic activity was investigated based on method proposed by Zhao et al. (2013). For this purpose, enzyme activity was assayed using the mixture including 25 μl of enzyme (0.5 mg/ml), 25 μl of β-pNPG (1, 5 mM), and 25 μl of glucose (1–10 M) that is incubated at 40°C for 10 min. In the absence of glucose, the initial activity of the enzymes was considered as 100%. Glucose inhibition constant (*K*_*i*_) of constructed and purified PersiBGL1 was examined utilizing the Dixon plot analysis (Zhao et al., 2018).

### Effect of Metal Ions and Chemical Reagents on β-Glucosidase Activity

The β-pNPG (10 mM) was added into the reaction mixture containing each of the metal ions including MgCl_2_, CaCl_2_, NaCl, MnCl_2_, CuSO_4_, FeSO_4_, ZnCl_2_, EDTA, Urea, PMSF, NaN_3_ (5 mM), and SDS, CTAB, Tween 20, and Triton X-100 at concentrations of chemical reagents (1%) with enzyme after being pre-incubated at 25°C for 30 min.

Various concentrations of NaCl (0.05, 0.1, 0.5, 1, 2, 3, 4, and 5 M) in 50 mM phosphate buffer (pH 8) were used to examine the salt tolerance of the enzyme. Meanwhile, the enzyme activity was evaluated under standard condition and the activity of control solution without the presence of metal ions and chemical reagents was assumed as 100% (Li et al., 2012).

### The Hydrolysis of Cellobiose by β-Glucosidase

The enzymatic hydrolysis of cellobiose (1% w/v) by PersiBGL1 was investigated by incubation of enzyme and cellobiose at 40°C in 50 mM phosphate buffer (pH 8). At different times, samples were collected and the amount of released glucose was measured in 24-h time intervals by glucose oxidase–peroxidase method using 3,3′,5,5′-tetramethylbenzidine (Foerster and Lynen, 1985) until 380 h.

## Results and Discussion

**FIGURE 1:**
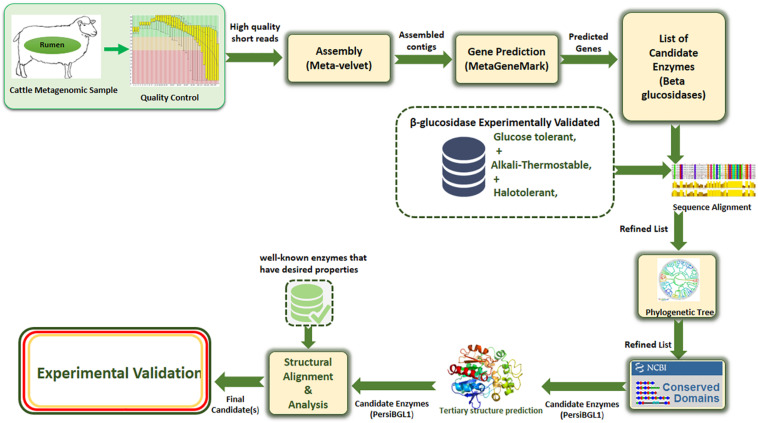
The proposed multi-stage workflow for identification of enzymes with desired properties from high-throughput sequencing data.

Our applied methodology in this study greatly reduced the amount of laboratory work through a more precise multi-step computational process. Assembling and screening raw metagenome data, predicting potential BGLs, identifying the BGL enzymes having all the desired features, selecting the most appropriate candidates, 3D modeling of the selected enzymes, and investigating the molecular mechanisms behind these important features, all done before starting the laboratory phase, result in highly targeted and highly successful laboratory tasks (Figure 1).

By this method, we isolate a BGL from sheep rumen metagenome, which shows high stability and activity in relatively high temperatures and alkaline pH. More importantly, it was notably insensitive to glucose as a competitive inhibitor, and even its catalytic activity soared by the addition of some ionic salt. This enzyme can substantially augment the conversion efficiency of agricultural waste to biofuel economically and industrially.

### Screening of PersiBGL1 by Sequence and 3D Structure Analysis

**FIGURE 2:**
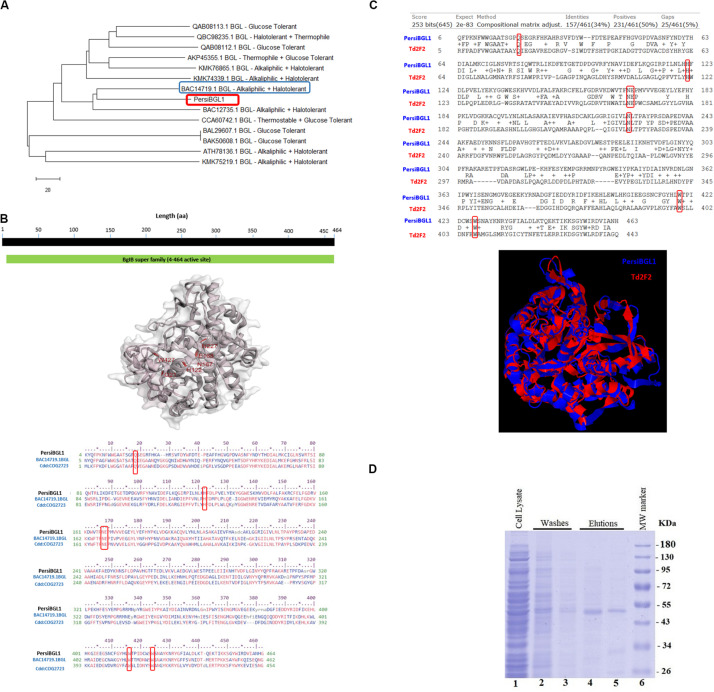
**(A)** The phylogenetic tree constructed with Neighbor Joining method. From an evolutionary perspective, the PersiBGL1 is most similar to two alkaliphilic and halotolerant BGL enzymes. **(B)** Predicted 3D structure of PersiBGL1 obtained from Phyre2 prediction server. The CDD revealed the active sites and alignment of PersiBGL1 and BAC14719.1 and COG2723. Red boxes show the region containing the catalytic domain in PersiBGL1, Gln18, His122, Asn167–Glu168, Trp416, and Trp424. **(C)** The sequence and structural alignment between the predicted pdb model for PersiBGL1 and Td2F2 performed by TM-Align. Red boxes show the conserved glucose binding residues. **(D)** SDS-PAGE and analysis of recombinant xylanase. SDS-PAGE was performed using a 12% polyacrylamide gel and stained with Coomassie Brilliant Blue. Lane 1 shows supernatant of *E. coli*’s lysis containing recombinant xylanase. Lanes 4–5, purified recombinant β-glucosidase. Lane 6, molecular weight of marker standard.

The PersiBGL1 was one of the most similar sequences to the 13 sequences extracted from literature that its cloning and expression procedures were done successfully. The phylogenetic tree constructed using 13 BGLs with known properties besides the PersiBGL1 is demonstrated in Figure 2A. From the set of 13 mentioned sequences, an alkaliphilic and halotolerant BGL from *Oceanobacillus iheyensis* HTE831 (accession number BAC14719.1) with E-value 1E-173 and Bit score 487 is the nearest neighbor of PersiBGL1 in the phylogenetic tree.

The CDD search revealed that the PersiBGL1 has a conserved 460 amino acids (AA) domain with PSSM-ID 225343 (Cdd:COG2723), Bit Score 584.60, and E-value 0e + 00. This is a Beta-glucosidase/6-phospho-beta-glucosidase/beta-galactosidase with carbohydrate transport and metabolism functionality. The active sites and alignment of PersiBGL1 and BAC14719.1 and COG2723 are shown in Figure 2B. The region containing the catalytic domain in PersiBGL1, Gln18, His122, Asn167-Glu168, Trp416, and Trp424 is well conserved.

Although there is a close identity between the PersiBGL1 and a β-glucosidase from *Selenomonas bovis* (NCBI Reference Sequence WP_164175414.1), due to uncharacterized properties of this enzyme, various experiments were performed to reveal the properties of the PersiBGL1 enzyme.

The phyre2 suggests that the double mutant (cys211ser/cys292ser) 6-2 phospho-b-d-glucosidase from *Lactobacillus plantarum* with PDB code 5NAV has the most similar tertiary structure to the PersiBGL1 with confidence score 100%.

By EzMol software (Reynolds et al., 2018), the 3D structure of PersiBGL1, including domains and the location of active sites was presented (Figure 2B).

The sequence and structural alignment between the predicted pdb model for PersiBGL1 and Td2F2 as a high glucose-tolerant BGL (PDB code 3WH5) were performed. Figure 2C illustrates the pairwise global alignment of these two BGLs. These two BGLs have only 34% sequence identity with Bit-score 253. The most prominent feature of PersiBGL1 is its ultra-high glucose tolerance. The mechanism of high glucose tolerance in BGLs was investigated by Matsuzawa et al. (2016). They identified the key role of an asparagine amino acid (Asn223) for glucose tolerance and substrate specificity in a Td2F2, a GH1 BGL with 3WH5 PDB code.

Figure 2C shows the graphical result of the structural alignment of these enzymes obtained from TM-Align. In contrast to sequence alignment, the structural alignment confirms very high similarity between the structures of PersiBGL1 and Td2F2. Among 465 residues of PersiBGL1, 93% of them aligned with Td2F2 residues with distance less than 5.0 $\overset{{}^{\circ}}{\text{A}}$. The RMSD value is 1.82 $\overset{{}^{\circ}}{\text{A}}$ and the TM-score is 0.90559 and 0.94719 if normalized by length of PersiBGL1 and Td2F2, respectively. The TM-score near to 1 indicates that both enzymes belong to the same structural folding class and the small RMSD indicates the very high structural similarity between two BGLs. The very close structural similarity between these two enzymes suggests that they should have also similar functional properties.

As it can be seen in Figure 2C, the Asn223 from Td2F2 is aligned with Asn227 in the PersiBGL1. The very high structural similarity between PersiBGL1 and a well-known glucose-tolerant enzyme besides the existence of the key asparagine amino acid in similar locations in both sequences strongly suggests that the PersiBGL1 should have high glucose tolerance features. The experiments described in the next subsections confirmed the ultra-high glucose tolerance in this novel BGL.

### Expression and Purification of PersiBGL1

The sequence of PersiBGL1 fragments was directly amplified from the metagenomic DNA of sheep rumen with degenerate primers and overexpressed under the control of the T7 promoter of pET-28a vector in *E. coli* BL21 (DE3). The recombinant enzyme was purified as the N-terminus His-tagged protein by using Ni-NTA Fast Start Kit and protein fractions from the purification steps were analyzed by SDS-PAGE (Maleki et al., 2020), and a single protein band was visible after the final purification step with a molecular weight to the calculated 53.92-kDa mass (Figure 2D).

#### Effects of pH and Temperature on the PersiBGL1 Activity

**FIGURE 3:**
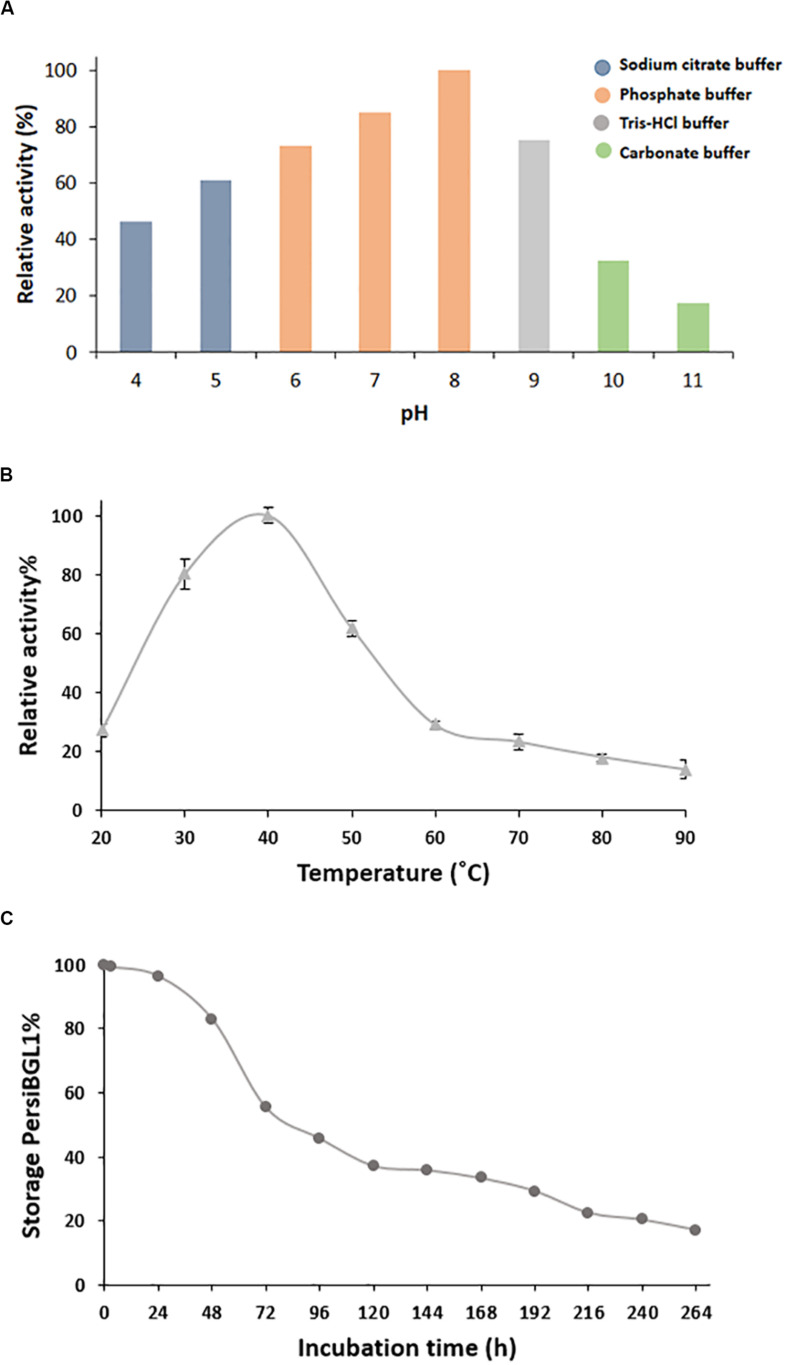
Characterization of purified PersiBGL1. **(A)** Effect of pH on PersiBGL1 activity. **(B)** Effect of temperature on PersiBGL1 activity. **(C)** The storage stability of PersiBGL1.

The temperature and pH are the most recognizable factors that play an instrumental role in enzyme stability. After purification of PersiBGL1, we deduced the effect of temperature and pH on catalyzing activity of the enzyme in Figure 3.

Bacterial BGLs isolated from thermophilic microorganisms depict enzyme activity mainly at pH less than 7. In contrast, PersBGL1 reached its maximum activity in pH 8 and retained more than 50% of its maximum value in the pH range between 6 and 10 (Figure 3A). All this evidence about pH feature of the PersiBGL1 substantiates its commercially valuable potential in biotechnological industries. Similarly, it has been reported that BGLs remaining active in broad range pH could ameliorate the version efficiency of cellulosic materials into glucose. Therefore, pH stability is another attribute that develops BGL productivity for marketing purposes (Jiang et al., 2011; Xia et al., 2016).

The enzymes with hydrolysis activity in the breakdown process of biomass need to be enhanced in an effort to reach higher stability at elevated temperatures (Colabardini et al., 2016).

In this case, BGLs belonging to the GH1 family are the most thermostable enzyme reported so far (Singhania et al., 2017). In Figure 3B, PersiBGL1 activity was measured in temperature ranging from 20 to 90°C in order to determine optimal temperature, which is 40°C. According to the F.2B, the enzyme relative activity has surged approximately 100% when the temperatures increased to 40°C.

However, PersBGL1 retained 80% of its relative activity after being stored for 48 h at 40°C. The enzyme activity was diminished drastically after 264 h of storage (Figure 3C).

### Kinetics Assay and Glucose Inhibition Constant

Although the mechanism of glucose inhibition effect on BGL activity is not entirely known, it is all transparent that BGL activity is frequently sensitive to glucose presence and decreases by the increased glucose concentration. Glucose inhibition limits the use of such BGs in industrial applications because an inefficient enzyme contributes toward biofuel costs. Therefore, BGLs are glucose tolerant in high demand.

**FIGURE 4:**
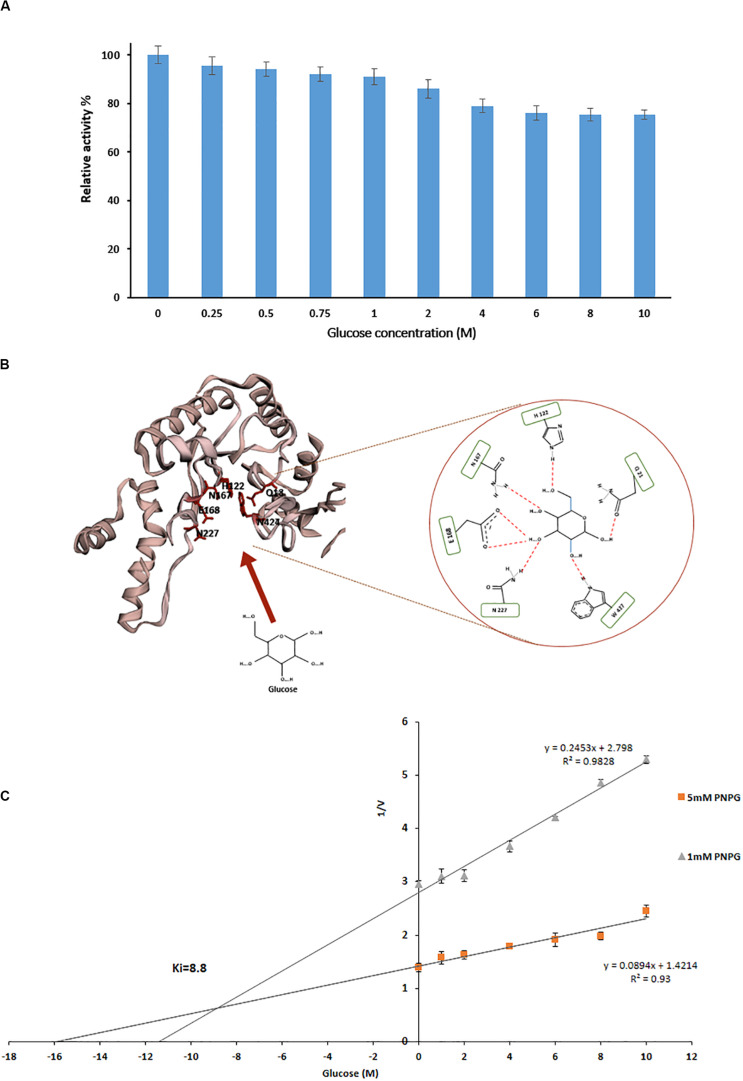
**(A)** Effect of different glucose concentration on relative activity of PersiBGL1. **(B)** The schematic of the glucose binding mechanism to the predicted structure of PersiBGL1. The Asn227 residue plays a key role in the glucose tolerance property of PersiBGL1 similar to Td2f2. **(C)** Dixon plot used for determination of *K*_*i*_ by measuring the purified PersiBGL1 against pNPG (1, 5 mM) in the presence of 0–10 M glucose in the reaction mixture.

Measurement of relative activity of PersiBGL1 in the presence of glucoses showed that with concentrations of less than 1 M, the enzymatic activity had no remarkable effect, and even in presence of 10 M glucose, it only had a little inhibition effect and PersiBGL1 retains 75% of its initial activity (Figure 4A).

The recombinant β-glucosidase, Td2F2 (Matsuzawa et al., 2016), which exhibited high glucose tolerance activity, was selected for further analytical studies to decipher the mechanism behind the high glucose tolerance property of PersiBGL1.

We compared the glucose binding site structure of the PersiBGL1 with the well-characterized GH1 BGL, Td2F2. The comparison reveals that almost all of the glucose binding site residues are conserved in both enzymes. Figure 4B shows the schematic view of the glucose binding mechanism to the predicted structure for PersiBGL1 and the involved residues. The important point is that, similar to Td2f2, the Asn227 residue (aligned with Asn223 in Td2f2) plays a key role in the glucose tolerance property of PersiBGL1.

Among reported BGLs that were derived from microorganisms, a vast proportion of them (with some exceptions) showed *K*_*i*_ values in the range of 0.2 and 4280 mM when the enzyme was assayed with pNPG, and a nice compilation of them could be found here (Li et al., 2012; Teugjas and Väljamäe, 2013; Sinha and Datta, 2016). The highest *K*_*i*_ values found in the literature was 4.28 M from soil metagenomic library (Li et al., 2012). In the same manner, we tested the glucose inhibition by assessing the influence of glucose on the kinetic parameters of PersiBGL1. Then, a *K*_*i*_ value of 8.8 M was determined by the Dixon plot (Figure 4C).

Several glucose-tolerant BGLs have been purified and characterized. Table 1 contains a list of BGLs across the GH1 and GH3 families with *K*_*i*_ of glucose when the enzyme was assayed with pNPG. Aside from the microbes isolated from metagenomes, studies have identified the rest in this list of microbial sources. In Table 1, PersiBGL1 depicted an extraordinary *K*_*i*_ value in comparison to other known BGL representatives. Bgl1269 with a *K*_*i*_ value of 4.28 M was presumed to have the highest tolerance to glucose compared to the BGLs discovered previously, while its *K*_*i*_ is less than half of the *K*_*i*_ measured in PersiBGL1 (8.8 M) (Li et al., 2012).

**TABLE 1:** The property comparison of PersiBGL1 with some known β-glucosidase representatives.

**Name**	**Source**	**Optimum pH**	**Ki**	**References**
Bgl6	Metagenomic library	5	3.5 M	Cao et al., 2015
Bgl1269	Soil metagenomic library	6	4.28 M	Li et al., 2012
Tt-BGL	Thermotoga thermarum DSM 5069T	4.8	1.5 M	Zhao et al., 2013
H0HC94	Agrobacterium tumefaciens	7.2	0.686 M	Sinha and Datta, 2016
TD2F2	Compost microbial metagenome	5.5	1M	Uchiyama et al., 2013
bgl1A	Marine microbial metagenome	6.5	1M	Fang et al., 2010
Unbgl1A	Soil metagenome	5	1.5 M	Lu et al., 2013
DturβGlu	Dictyoglomus turgidum	5.4	0.7M	Limauro et al., 2018
BglD5	Jeotgalibacillus malaysiensis	7	2.5 M	Chan et al., 2018
PersiBGL1	Rumen metagenome	8	8.8 M	This study

*Ki measured for glucose with pNPG as substrate.*

Implementing the Lineweaver–Burk map and pNPG as substrate enabled us to assess the kinetic parameters (*k*_*m*_ and *V*_*max*_) corresponding to the activity of PersiBGL1. The kinetic constants (*k*_*m*_ and *V*_*max*_) corresponding to the activity of PersiBGL1 can be comprehensively determined and measured if the Lineweaver–Burk plot is used. The *k*_*m*_, *V*_*max*_, *k*_*cat*_, and *k*_*cat*_/*k*_*m*_ values of the enzyme were estimated to be 1.25 mM, 1.55 mM/min, 3.1 min^–1^ and 2.47 min^–1^ mg^–1^ ml, respectively.

Consequently, to our knowledge, PersiBGL1 displays the highest inhibitor constant (*K*_*i*_) among all reported BGLs. Additionally, PersiBGL1 revealed higher *V*_*max*_ (1.55 mM/min) in comparison to some other known BGL representatives, which indicates that PersiBGL1 possesses better turnover number or catalytic rate than some enzymes brought in Table 1. A considerably high glucose inhibition resistance and a decent catalytic rate of PersiBGL1 clearly distinguish this enzyme from many other identified BGLs for cellulolytic production purposes.

### Effect of Metal Ions and Chemical Reagents on Enzyme Activity

It has been proposed that metal ion could be responsible for impeding the catalytic activity of BGL (Chen et al., 2014). Given this fact, exploiting a BGL that is non-sensitive to salt or to some degree can tolerate the presence of metal ion in solutions is remarkably beneficial.

**FIGURE 5:**
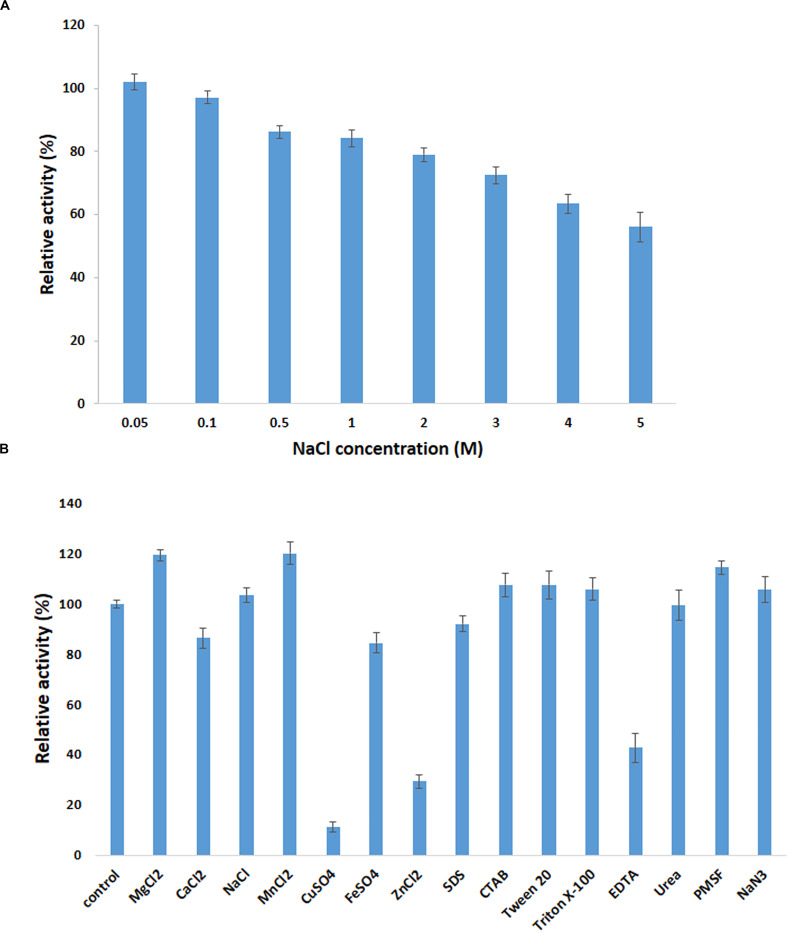
Effect of metal ions on PersiBGL1 activity when enzyme treated with different concentrations of salts compared to control condition without the presence of salt. **(A)** The presence of different concentrations of NaCl. **(B)** The metal ions, chemical agents, and detergents.

The influence of various metal ions and chemicals on the activity of PersiBGL1 was investigated by using pNPG as a substrate and standard assay procedure (Figure 5). The control reaction was carried out under the same conditions only without the presence of metal ions and chemical reagents.

It can be readily observed that the enzyme could maintain its activity in the presence of all the metal ions except Cu^2+^ and Fe^2+^, which inhibited the activity of PersiBGL1 to 11.13 and 29.27%, respectively. On the other hand, the maximum relative activities among the metal ions were obtained by MnCl_2_ (120.07%) followed by MgCl_2_ (119.30%) and NaCl (103.36%).

The addition of the different surfactants and detergents showed no observable change in the activity of the enzyme. Among the surfactants, only the presence of the EDTA showed a negative effect on the action of PersiBGL1 (42.74%). Reduction of the enzymatic activity by the metal-chelating EDTA could represent the fact that PersiBGL1 could be a metal-dependent enzyme.

The salt tolerance of the PersiBGL1 was tested by measuring the activity in the presence of various concentrations of NaCl (Figure 5). Results indicated that the enzyme could maintain its activity at high and low concentrations of NaCl. The relative activities slightly decreased by the increment of salt concentration. At the highest concentration of NaCl (5 M), the PersiBGL1 demonstrated near 60% of activity, which confirmed the salt tolerance of the β-glucosidase. Thus, in addition to thermostability and glucose tolerance significance in biofuel, this feature is another notable characteristic of PersiBGL1 that is worth mentioning.

### Effect of BGLs on Enzymatic Saccharification of Cellobiose Hydrolysis

Cellulose, a naturally occurring linear polysaccharide, is a substantial structural component of the primary cell wall in green plants. Cellulose, as a renewable feedstock, is ranked first among all bio-polymers, and its potential for conversion to ethanol and other chemicals has made it one of the main promising green alternatives to fossil fuels. However, the efficient bioconversion of lignocellulosic biomass into biofuels in general and the saccharification of cellobiose as the most critical stage of this conversion in particular remain a major challenge in cellulose degradation. The cellobiose, a disaccharide reducing sugar, needs a BGL to be converted to glucose in the final step of cellulose hydrolysis and had important applications in the feed, food, biofuel, and other fields (Pogorzelski and Wilkowska, 2007; Song et al., 2011; Sun et al., 2014). One of the efficient strategies to circumvent this challenge is to search for a BGL with high glucose tolerance, thermostability, and high catalytic efficiency in harsh environments. The rumen environment is a good example of such environments, and we applied a metagenomic approach to screen this BGL.

**FIGURE 6:**
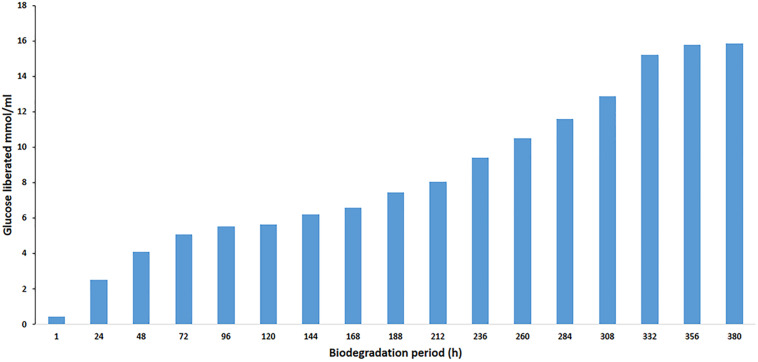
Curves indicate the amount of glucose yield in enzymatic hydrolysis of cellobiose with PersiBGL1 after 380 h of biodegradation period.

The effect of BGL on the liberation of glucose during the degradation of cellobiose was studied. To evaluate enzyme efficiency in cellobiose hydrolysis, cellobiose (1% w/v) was hydrolyzed at 40°C in phosphate buffer (50 mM, pH 8). The enzyme/substrate ratio was 1 mg/g cellobiose (1:1000). In the hydrolysis process, the amount of used PersiBGL1 was 0.1 μg. The final liberated concentration of glucose is 15.85 mM and the rate of conversion reaches to zero after 380 min, while 25% of cellulose was hydrolyzed at this point (Figure 6).

Similarly, spontaneous increases in the concentration of glucose liberated during the degradation period from 1 h (0.44 μmol/ml) to 380 h were observed, yielding maximum release of glucose (15.85 μmol/ml). Glucose production did not decrease significantly after 300 h. Hence, this result suggests that this novel BGL could be a suitable enzyme in the saccharification of lignocellulosic biomass.

## Conclusion

In this study, the sheep rumen metagenome was explored using computational screening for finding a novel stable BGL with a significantly very high tolerance to glucose. The main advantage of applying our proposed computational method is that instead of simple alignment-based screening, a multi-step procedure is employed to refine a vast amount of potential BGLs and narrow down the list of candidates to a very limited number of enzymes with required properties. In this approach, the relatively heavy cost of functional screening is replaced by computational costs. This approach uses an eight-stage procedure including quality control, assembly of metagenomic short reads, gene prediction, filtering enzymes based on sequence similarity with known enzymes mined from literature, determining phylogenetic position of candidates, finding conserved domain related to the desired enzymatic activity, prediction of tertiary structure of candidate enzymes, and performing structural alignment with the best-known enzymes to find an appropriate candidate from metagenomic data and ensure that the final candidates have desirable properties. Applying this approach is not limited to BGLs and certainly can be used in a wide domain of enzymatic applications in order to identify novel natural enzymes, suitable for specific industrial applications.

After identification, cloning, expression, purification, and characterization of PersiBGL1, the accuracy of computational prediction was experimentally confirmed. The PersiBGL1 is a halotolerant stable enzyme with distinguished resistance to high concentration of glucose. It was successfully utilized for the saccharification of cellobiose.

## Data Availability Statement

The datasets presented in this study can be found in online repositories. The names of the repository/repositories and accession number(s) can be found in the article/Supplementary Material.

## Author Contributions

SA: conceptualization, methodology, supervision, investigation, writing - original draft, resources, and project administration. SN-N, MR, and MeM: biochemical characterization and analyzed and interpreted the data. KK: conceptualization, methodology, contributed to the computational and bioinformatics data analysis, writing - review and editing. AP and MaM: performed the gene cloning, expression, and purification. SM: investigation, resources, and writing - original draft. GS: supervision, resources, writing - review and editing, project administration, funding acquisition, and conceptualization. All authors contributed to the article and approved the submitted version.

## Conflict of Interest

The authors declare that the research was conducted in the absence of any commercial or financial relationships that could be construed as a potential conflict of interest.
