# Supplementary material for: A Novel High Glucose-Tolerant β-Glucosidase: Targeted Computational Approach for Metagenomic Screening
Source: Front Bioeng Biotechnol. 2020 Jul 30;8:813. doi: 10.3389/fbioe.2020.00813 (PMC7406677; doi:10.3389/fbioe.2020.00813)
Supplement: Supplementary file 1 [file Table_1.DOCX]

**Metagenomic data and the analysis protocol of sheep's rumen microbiota**

Collected samples:

The Kochia (Kochia scoparia, KS) as lignocellulosic biomass was selected for in sacco rumen incubation and degradation analysis. Three adult male fistulated Shal (Iranian native) sheep of body weight between 62-68 kg were used for the study. The animals were housed in a barn and fed a diet of 70% wheat straw and 30% barley-based ad libitum twice daily at 08:00 and 16:30. The animals had free access to drinking water. Feeds were introduced into the rumen via the fistula; the material was first baked at 55°C for 48 h in an air-circulating oven and was then ground finely enough to pass through a 2 mm sieve.

An aliquot of this ground material (5 ± 0.05 g) was packaged within a heat-sealed nylon bag (5 × 10 cm; 50 μm pore size). The feed samples were incubated in duplicate per sheep and replicated in the three animals, which were placed within the rumen shortly after a morning meal; two bags per each feed were removed after 24 h, and a further two after each of 48 h, 72 h, and 96 h. After removal, one of the pair of bags was severely washed in order to analyze fiber breakdown, while the other was rinsed in distilled water, then gently squeezed to remove unattached or loosely attached microbes. The bags were then snap-frozen in liquid nitrogen and stored at -80°C until required for analysis. The contents of a non-incubated (0 h) bag were used as a control for the degradation analysis.

Extraction of metagenomic DNA:

Microbial cells adhering to the digested material were recovered by suspending 8–10 g (wet weight) of the digested feed particulates in 20 mL 0.1% (v/v) Tween 80, 1% (v/v) methanol and 1% (v/v) tertiary butanol (pH 2), following Pope et al. [17]. After a 1-3 min vortex, the plant material was sedimented by imposing three rounds of low speed centrifugation, with the supernatant being transferred to a new tube after each spin. Finally, the microbial content was recovered by a rapid centrifugation (12,000 g for 5 min). Microbial DNA was extracted using the QIAamp® DNA Stool Mini Kit (Qiagen, Hilden, Germany). The concentration of the DNA preparations was determined using a NanoDrop spectrophotometer (Thermo Scientific, Wilmington, DE, USA) and its integrity was checked by inspection of an electrophoretically separated (1% agarose gel) aliquot.

Sequencing technique for metagenome:

Metagenome library preparation was conducted using the TruSeq DNA Library Preparation Kit (Illumina, San Diego, CA, USA), and the quantity of each library was evaluated using a Qubit fluorimeter (Invitrogen, Carlsbad, CA, USA).

Metagenome sequencing was performed at the Novogene Inc. (Beijing, China) using Illumina HiSeq 2500 sequencing platform with 150 bp paired-end sequencing of ~ 340 bp fragments.

NCBI BioProject database:

Raw sheep rumen metagenomic data was submitted to NCBI with Bioproject ID: PRJNA635543.

The assembly software and applied parameters:

All metagenome sequences were co-assembled using MEGAHIT v1.2.9, with the following options: --k-min 31, --k-max 141, --k-step 10, --min-count 2 and --min-contig-len 200

| The assembly statistics:  # 356402 sequences of average length 335.96 |
| --- |
| # total length 119738111 |
| # minimum length 20 |
| # maximum length 7776 |
| # distribution of sequence length in buckets of size 500 |
| 0--499 293516 |
| 500--999 55707 |
| 1000--1499 6025 |
| 1500--1999 768 |
| 2000--2499 187 |
| 2500--2999 86 |
| 3000--3499 57 |
| 3500--3999 29 |
| 4000--4499 16 |
| 4500--4999 7 |
| 5000--5499 1 |
| 5500--5999 1 |
| 6000--6499 1 |
| 7500--7999 1 |
| # number of contigs: 356402 |
| # total contigs length: 119738111 |
| # mean contig size: 335.96 |
| # contig size first quartile: 171 |
| # median contig size: 288 |
| # contig size third quartile: 435 |
| # longest contig: 7776 |
| # shortest contig: 20 |
| # contigs > 500 nt: 62558 (17.55 %) |
| # contigs > 1K nt: 7154 (2.01 %) |
| # contigs > 10K nt: 0 (0.00 %) |
| # contigs > 100K nt: 0 (0.00 %) |
| # contigs > 1M nt: 0 (0.00 %) |
| # N50 427 |
| # L50 93229 |
| # N80 258 |
| # L80 200225 |
