# Supplementary material for: A Novel High Glucose-Tolerant β-Glucosidase: Targeted Computational Approach for Metagenomic Screening
Source: Front Bioeng Biotechnol. 2020 Jul 30;8:813. doi: 10.3389/fbioe.2020.00813 (PMC7406677; doi:10.3389/fbioe.2020.00813)
Supplement: Supplementary file 2 [file Table_2.DOCX]

**13 BGL enzymes obtained from literature mining with at least one of the glucose tolerance, halotolerance, thermophilic, or alkaliphilic properties.**

|  | GenBank: | Properties | DOI |
| --- | --- | --- | --- |
| 1 | QAB08113.1 | Glucose Tolerant 1M  Highest activity temp.=50C  Stable in pH:4.0-9.0  Highly glucose tolerant | DOI:10.3389/fmicb.2018.03149 |
| 2 | QBC98235.1 | salt- and glucose and xylose tolerant Ki for glucose = 932 mM | DOI:10.1007/s13399-019-00556-5 |
| 3 | QAB08112.1 | Glucose-Tolerant  The inhibition constants (Ki) of AaBGL1 and AaBGL2 were 1502 and 193.5 mM glucose | DOI:10.3389/fmicb.2018.03149 |
| 4 | AKP45355.1 | Glucose-Tolerant  Ki of 800 mM glucose. | DOI: 10.1007/s00253-015-6619-9 |
| 5 | KMK76865.1 | Alkaliphilic Halotolerant | DOI:10.1128/genomeA.00919-15. |
| 6 | KMK74339.1 | Alkaliphilic Halotolerant | DOI:10.1128/genomeA.00919-15 |
| 7 | BAC14719.1 | Extremely Halotolerant and Alkaliphilic | - DOI:10.1093/nar/gkf526 |
| 8 | BAC12735.1 | Extremely Halotolerant and Alkaliphilic | DOI:10.1111/j.1574-6968.2001.tb10963.x. |
| 9 | CCA60742.1 | thermostable and glucose-toleran  a broad temperature range (80–100°C).  Ki value of 211 mM | DOI 10.1007/s00253-011-3406-0 |
| 10 | ATH78136.1 | Alkaliphilic Halotolerant  maximum activity at pH 8.5 | DOI 10.1007/s00792-016-0852-8 |
| 11 | KMK75219.1 | Alkaliphilic Halotolerant | doi:10.1128/genomeA.00919-15 |
| 12 | BAL29607.1 | glucose-tolerant | doi.org/10.1093/dnares/dsr042 |
| 13 | BAK50608.1 | glucose-tolerant | doi:10.1093/dnares/dsr042 |
