# Supplementary material for: A Novel High Glucose-Tolerant β-Glucosidase: Targeted Computational Approach for Metagenomic Screening
Source: Front Bioeng Biotechnol. 2020 Jul 30;8:813. doi: 10.3389/fbioe.2020.00813 (PMC7406677; doi:10.3389/fbioe.2020.00813)
Supplement: Supplementary file 3 [file Table_3.DOCX]

LOCUS BSeq#1 1398 bp DNA linear ENV 03-JUN-2019

ACCESSION MN016943 (will be available from 2021)

SOURCE plastid uncultured bacterium

ORGANISM uncultured bacterium

Bacteria; sheep rumen sample.

gene 1..1398

/gene="PersiBGL1"

/allele=" PersiBGL1"

CDS 1..1398

/gene=" PersiBGL1"

/allele=" PersiBGL1"

/codon_start=1

/product=" PersiBGL1"

/translation="MAVKYQFPKNFWWGAATSGPQSEGRFHKAHRSVFDYWFDTEPEA

FFHGVGPDVASNFYNDYTHDIALMKCIGLNSVRTSIQWTRLIKDFETGETDPDGVRFY

NAVIDEFLKQGIRPILNLHHFDLPVELYEKYGGWESKHVVDLFALFAKRCFELFGDRV

KDWVTFNEPMVVVEGEYLYEFHYPKLVDGKKACQVLYNLNLASAKAIEVFHASDCAKL

GGRIGIVLNLTPAYPRSDAPEDVAAAKFAEDYKNNSFLDPAVHGTFTEDLVKVLAEDG

VLWESTPEELEIIKNHTVDFLGINYYQPFRAKARETPFDASRGWLPEKHFESYEMPGR

RMNPYRGWEIYPKAIYDIAINVRDNLGNIPWYISENGMGVEGEEKYRNADGFIEDDYR

IDFIKEHLEWLHKGIEEGSNCFGYHLWTPIDCWSWSNAYKNRYGFIALDLKTQEKTIK

KSGYWIRDVIANHGF"

BASE COUNT 313 a 408 c 396 g 281 t

ORIGIN

1 atggcagtca agtatcagtt cccgaagaat ttctggtggg gcgcggcgac gtccggaccg

61 cagtccgagg gacgtttcca caaggcacac agaagcgttt ttgattattg gttcgatacg

121 gagccggagg cattctttca cggtgtcggc ccggatgtcg cgtcgaattt ctataatgac

181 tacacgcatg acatcgcgct catgaagtgc atcggtctga actccgtgcg tacatccatt

241 cagtggacgc gcctcatcaa ggacttcgag acgggcgaga cggatcccga cggtgtgcgg

301 ttctacaacg ccgtcatcga tgagttcctc aagcagggca tccgccctat cctgaacctc

361 catcacttcg atctgcccgt cgagctctat gaaaaatacg gcggctggga gtcaaaacac

421 gtcgtcgacc tcttcgcgct tttcgcgaag cgttgcttcg agctcttcgg cgaccgtgtc

481 aaggactggg tcacgttcaa cgagccgatg gtcgtcgtcg agggcgagta tctctacgag

541 ttccattatc cgaagctcgt cgacgggaag aaagcctgcc aggtgctcta taacctcaac

601 ctcgcctcgg cgaaagccat cgaggtgttc catgcgtccg actgcgcgaa gctcggcggc

661 cgcatcggca tcgtgctgaa cctcacgccg gcctacccgc gttccgacgc gccggaggac

721 gtcgccgcgg cgaaattcgc ggaagattac aaaaacaatt cattcctcga ccctgccgtg

781 cacggcacgt tcactgagga tctcgtcaag gtgctcgcgg aggacggtgt gctctgggag

841 tcgacgccgg aggagcttga gatcatcaag aaccatacgg tcgatttcct cggcatcaac

901 tactatcagc cgttccgcgc gaaggcgcgc gagacgccgt tcgatgcgag ccgcggctgg

961 ctgccggaga agcatttcga aagctacgag atgccgggac gccgcatgaa cccgtaccgc

1021 ggctgggaga tctatccgaa ggccatctac gacatcgcca tcaacgtgcg cgacaatctc

1081 ggcaacatcc cctggtacat ctctgagaac ggcatgggcg tcgagggcga ggagaaatac

1141 cgcaacgccg acggcttcat cgaggacgac taccgcatcg acttcatcaa ggagcatctt

1201 gagtggctgc acaagggtat cgaggaagga tcgaactgct tcggctatca cctctggacg

1261 ccgatcgact gctggtcctg gtcgaacgct tacaagaacc gctacggctt catcgcgctc

1321 gacctcaaaa cacaggaaaa aaccattaaa aaatcaggct attggatccg cgatgtcatt

1381 gcgaatcatg gattttga

//
